# Colchicine-Resistant Familial Mediterranean Fever With Depressive State Successfully Treated With Escitalopram

**DOI:** 10.7759/cureus.15145

**Published:** 2021-05-20

**Authors:** Masamitsu Toshida, Yuki Konishi, Atsuko Ikenouchi, Naomichi Okamoto, Reiji Yoshimura

**Affiliations:** 1 Psychiatry, University of Occupational and Environmental Health, Kitakyushu, JPN

**Keywords:** familial mediterranean fever, depressive state, escitalopram, colchicine, prednisolone

## Abstract

Familial Mediterranean fever (FMF) is a hereditary autoinflammatory disease associated with the Mediterranean fever (MEFV*)* gene and is mainly characterized by periodic fever and serositis. Colchicine has been used to prevent FMF episodes and reduce the frequency of attacks. We report the case of a 64-year-old man who presented with depressive symptoms and was resistant to colchicine treatment. Adding escitalopram to the ongoing colchicine regimen dramatically improved his fever, abdominal pain, and depressive symptoms. The change in cytokines, ABCB1 effects, and increased serotonin were related to these mechanisms. This case suggested that adding escitalopram to colchicine is a viable treatment option for colchicine-resistant FMF.

## Introduction

Familial Mediterranean fever (FMF) is characterized by recurrent acute fever, peritonitis, pleurisy, and arthritis with high inflammatory mediators. Acute attacks usually resolve within two to four days. However, even during the asymptomatic phase, these inflammatory mediators may be higher than normal, and the inflammation persists [1]. The Mediterranean fever gene (MEFV) was identified as a disease-associated gene, but the mechanism behind its pathogenesis remains unclear [2]. The protein encoded by the MEFV gene is called pyrin. Consisting of 781 amino acids, pyrin is expressed in granulocytes, eosinophils, activated monocytes, fibroblasts of the plasma, and synovial membranes [3]. Pyrin acts as an inhibitory molecule in inflammation by regulating IL-1β and NF-κB activation [4]. Dysfunction and disruption of pyrin mainly cause FMF. Colchicine has been used to prevent FMF episodes, and reduce the frequency and severity of attacks [5]. IL-1 receptor antagonists and anti-NTF-α have also been used in colchicine-refractory cases [6]. Selective serotonin reuptake inhibitors (SSRIs) were reportedly useful adjuncts in managing the FMF patients who continue to have attacks despite regular colchicine intake [7] because stress and emotional status can trigger FMF episodes. This sheds light on the role of cytokines in the pathogenesis of depression. We report the case of a patient with colchicine-resistant FMF and major depression secondary to a general medical condition. In this case, escitalopram significantly ameliorated the FMF attacks accompanied by depressive symptoms.

## Case presentation

**Figure 1:**
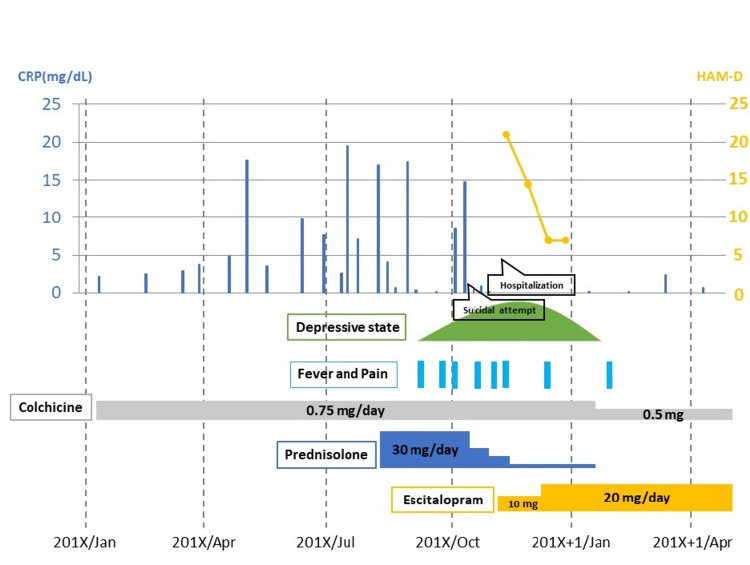
The time course of symptoms and medications in the case.

A 65-year-old male with frequent episodes of abdominal pain and fever was referred to the University of Occupational and Environmental Hospital. Approximately seven years before the consult, the patient experienced abdominal pain and high-grade fever, lasting for a few days and spontaneously resolving without treatment. Similar attacks were noted once every two months, but he did not seek medical care because the symptoms were tolerable and spontaneously relieved within a few days. During the first visit, the patient presented with a fever of approximately 38°C, abdominal pain, watery diarrhea, and constipation. His blood test results revealed a white blood cell count of 11640/μL, serum C-reactive protein (CRP) level of 14.7 mg/dL, and erythrocyte sedimentation rate of 59 mm/h. These findings suggested ongoing inflammation. He was diagnosed with FMF based on the Livneh criteria [8]. Exon 10 mutations were not found, but P369S and R408Q were present. Subsequently, he was treated with colchicine (0.75 mg/day) for three years. However, the FMF attacks persisted. Thus, prednisolone (30 mg/day) was added to his treatment. The FMF attacks were slightly relieved, and the dose of prednisolone was gradually increased. However, the patient began exhibiting symptoms of depressed mood, anxiety, insomnia, fatigue, and decreased motivation. Prednisolone was tapered off due to prednisolone-induced depression. However, his depressive mood and restlessness worsened and were accompanied by agitation and suicidal thoughts. He attempted suicide via hanging, failed, and was admitted to the psychiatry ward at our university hospital. He was considered to be in a major depression secondary to a medical condition given in the Diagnostic and Statistical Manual of Mental Disorders 5th Edition. His 17-item version of the Hamilton Assessment Scale for Depression (HAM-D) score was 22 points on admission. Escitalopram (10 mg/day) was added to the colchicine regimen and increased to 20 mg/day. His psychiatric symptoms drastically improved, and the HAM-D score decreased to seven points three weeks after escitalopram treatment. In addition, he also tested negative for CRP, and the fever and abdominal pain attacks completely disappeared. Since then, his physical FMF symptoms and accompanying depressive symptoms have not relapsed with escitalopram and colchicine. Written informed consent was obtained from the patient for this case report (Figure 1).

## Discussion

The present case showed that adding escitalopram to colchicine effectively alleviated FMF episodes of pain, fever, and depression. This case was consistent with previous cases showing that SSRIs, including paroxetine and fluoxetine, improved the disease activity of colchicine-refractory FMF [7]. However, the patient was administered low doses of colchicine combined with escitalopram in this case.

Patients with FMF have chronic physical symptoms that contribute to psychological stress associated with the development of depressive symptoms. As a result, inflammatory cytokines are elevated [8]. A depressive state and FMF synergistically activate the production of inflammatory cytokines [9]. SSRIs are efficacious against FMF because of their immunomodulatory effects. SSRIs, including escitalopram, alter the cytokine levels in major depression [10]. ABC transporter subfamily B member 1 (ABCB1) is responsible for the efflux of various drugs, including colchicine, out of the cell, and is associated with ABCB1 gene polymorphism and colchicine response in FMF patients [11]. Escitalopram also influences ABCB1 and affects the dose needed to achieve remission in major depression secondary to ABCB1 gene polymorphism [12]. Based on the findings in this case, the interaction between escitalopram and colchicine via ABCB1 likely contributed to their effects in a pharmacogenetic mechanism. However, ABCB1 genotyping was not performed. Moreover, plasma and platelet serotonin levels were reportedly involved in the disease activity of FMF. The plasma and platelet levels of serotonin were reduced during the attack [13]. Escitalopram increases the plasma and platelet serotonin levels induced by blocking serotonin transporters. In short, both pharmacokinetic mechanisms and pharmacodynamics of antidepressants might underlie the response seen in the patient. It has been reported that SSRIs have anti-inflammatory and immunomodulatory properties [14, 15]. The detailed mechanisms of action of SSRIs with colchicine need further investigation. Prednisolone was likely related to the occurrence of depressive symptoms, but the depressive symptoms persisted despite reducing the dosage of prednisolone. Thus, we cannot rule out the possibility that tapering off prednisolone improved depressive symptoms in this case.

## Conclusions

This case suggested that a possibility of adding escitalopram to low-dose colchicine treatment significantly improved FMF attacks accompanied by depressive symptoms.
